# INFANT FEEDING IN THE FIRST TWO YEARS OF LIFE

**DOI:** 10.1590/1984-0462/;2018;36;2;00004

**Published:** 2018

**Authors:** Wanessa Casteluber Lopes, Fúlvia Karine Santos Marques, Camila Ferreira de Oliveira, Jéssica Alkmim Rodrigues, Marise Fagundes Silveira, Antônio Prates Caldeira, Lucinéia de Pinho

**Affiliations:** a Faculdades de Saúde Ibituruna, Montes Claros, MG, Brasil.; b Universidade Estadual de Montes Claros, Montes Claros, MG, Brasil.

**Keywords:** Breast Feeding, Supplementary Feeding, Infant nutrition, Aleitamento materno, Alimentação complementar, Nutrição infantil

## Abstract

**Objective:**

To analyze the prevalence of breastfeeding and the introduction of complementary food for zero to 24-month-old infants.

**Methods:**

This is a population-based cross-sectional study of children aged less than 24 months in Montes Claros, Minas Gerais, Brazil. Data were collected in 2015, by interviews with people in charge of infant care in the house. The questionnaire administered assessed the sociodemographic status of the family, maternal and infant characteristics and food consumption habits. Survival analysis was used to calculate median prevalence and duration of breastfeeding and the introduction of complementary feeding.

**Results:**

With 180 days of life, 4.0% of the children were exclusively breastfed, 22.4% were mostly breastfed and 43.4% were fed breast milk as complementary food. In the third month of life, children were consuming water (56.8%), fruit juice or formula (15.5%) and cow’s milk (10.6%). At the age of 12 months, 31.1% were consuming artificial juice and 50.0% were eating candies. Before the age of 1 year, 25.0% of them had already eaten instant noodles.

**Conclusions:**

The introduction of drinks, honey, sugar and candies as complementary food was found to be premature; and solid and semi-solid foods were almost appropriate. The habits described can directly affect the success of breastfeeding. Given that the inadequate eating practices identified can compromise the infant’s health, actions that promote breastfeeding and provide guidance on the introduction of complementary foods are important*.*

## RESUMO

**Objetivo:**

Avaliar a frequência do aleitamento materno e a introdução da alimentação complementar em crianças de zero a 24 meses.

**Métodos:**

Estudo transversal de base populacional, composto por amostra representativa de crianças menores de 24 meses da cidade de Montes Claros, Minas Gerais. A coleta de dados foi realizada em 2015 por meio de entrevista, realizada nos domicílios dos participantes, com os responsáveis pelas crianças. Aplicou-se um questionário para avaliar a situação sociodemográfica da família, as características materno-infantis e o consumo alimentar. A análise de sobrevivência foi utilizada para calcular a prevalência e a duração mediana dos padrões de aleitamento materno e introdução da alimentação complementar.

**Resultados:**

Ao completarem 180 dias de vida, 4,0% das crianças estavam em aleitamento materno exclusivo, 22,4%, em aleitamento materno predominante, e 43,4%, em aleitamento materno complementar. As crianças já recebiam água (56,8%), suco natural/fórmula infantil (15,5%) e leite de vaca (10,6%) no terceiro mês de vida. Aos 12 meses de idade, o suco artificial foi oferecido para 31,1% das crianças e 50,0% já consumiam doces. E antes de completar um ano de idade, 25,0% das crianças já haviam consumido macarrão instantâneo.

**Conclusões:**

A introdução da alimentação complementar mostrou-se precoce para líquidos, mel, açúcar e guloseimas, próxima da adequação para alimentos sólidos e semissólidos, o que pode afetar diretamente o sucesso do aleitamento materno. As práticas alimentares inadequadas identificadas são capazes de comprometer a saúde da criança; por isso, ressalta-se a importância de realizar ações para a promoção do aleitamento materno acompanhadas de orientações para a introdução da alimentação complementar*.*

## INTRODUCTION

The first years of life of an infant are characterized by the speed of growth and development, and eating habits play an essential role to make sure that such phenomena take place adequately.^1^ ^,^ ^2^ The quality and the quantity of foods consumed by the child are critical aspects and have repercussions throughout life, being associated with the profile of health and nutrition, since childhood is one of the most biologically vulnerable stages of life considering nutritional deficiencies and disorders.^3^ ^,^ ^4^ ^,^ ^5^

The World Health Organization (WHO) recommends that the child be fed exclusively with breastmilk until the age of six months,^6^ which has a positive impact on the survival rate and on health at this phase and in adult life.^7^ Maternal milk contains the proper energy and nutrients for the degree of physiological maturity of the infant, besides protective factors against diseases, which makes it ideal for the first months of life.^5^ ^,^ ^7^ After the age of six months, it is important to begin complementary feeding, once the quantity and the composition of maternal milk are no longer sufficient to meet the nutritional needs of the infant.^8^

The inadequate introduction of foods in the diet of the infant may lead to damaging consequences to health, especially when the offer is made before the complete physiological development.^1^ Regarding the nutritional aspect, it is unfavorable, since it increases the risk of contamination and allergic reactions, interferes in the absorption of important nutrients in maternal milk, and implies in the risk of early weaning. On the other hand, the late initiation of foods is disadvantageous, since after the sixth month of life maternal milk no longer meets the energy needs of the child, leading to the deceleration of growth and increasing the risk of nutritional deficiency.^8^

There are few studies in Brazil about the pattern of complementary food introduction, with probability samples of children^9^ in the different regions of the country, which assess the survival free of inadequate foods. This study aimed at assessing the frequency of breastfeeding and the introduction of complementary foods in children aged from zero to 24 months.

## METHOD

This is a population-based, cross-sectional study, conducted in 2015 in Montes Claros, Minas Gerais, which is the main urban pole in the North region of the State. The target-population was composed by children aged less than 24 months living in the urban area of the city of the study.

The sample size was established based on a conservative 50% estimation for the prevalence of the studied event (early weaning), considering a 5% error and the correction factor for the sampling design (“*deff*”) equal to 1.5. There was also an addition of 10% to compensate for possible losses. The calculations showed the need for participation of at least 427 individuals.

A probability sample of permanent private households (PPH) in the urban zone was used, selected in two stages (census sector and blocks). In the first one, 64 census sectors were chosen systematically among the 385 that are present in the Geographic Operational Base (GOB), from 2010, in the Brazilian Institute of Geography and Statistics (IBGE). In the second one, in each census sector, the blocks that would be visited were selected randomly, including, in data collection, all children in the households aged less than 24 months. When the selected residence did not have children in the age group of the study, a new selection of households was conducted, according to the order of the precious selection.

A properly trained and calibrated team collected the data by interviewing the people in charge of the children in the households. The instrument of data collection included questions about the demographic situation of the Family (age and color of maternal skin, maternal schooling and occupation, marital status, Family income and parity), besides information about prenatal care, type of delivery, characteristics of the child (sex, weight at birth), participation in dietary supplement programs (vitamin A and ferrous sulfate), and analysis of dietary intake (consumption and frequency of breast milk, cow’s milk, infant formula, cereals, sugar, honey, chocolate milk, fruits, fruit juice, artificial juice, vegetables, beans, meat, instant noodles and junk food).

For the evaluation of maternal breastfeeding, the terminology used in this text was that proposed by the WHO:

- AME: use of human milk as the only food for the child.
- Predominant breastfeeding (PBF): use of human milk as the main source of nutrition, allowing the use of other liquids (water, juice or tea).
- Complementary foods: use of human milk associated with other foods, dairy or not, solid or liquid.
- Early weaning: introduction of complementary foods before the age of six months, interruption AME or PBF before this period.

Descriptive statistics, with absolute frequency (*n*) and percentage (%) values, were used to characterize the sample. In the description of proportions, the 95% confidence interval (95%CI) was calculated, and, to analyze the breastfeeding and food introduction, survival curve rates were elaborated. The statistical treatment of information was conducted using the software Statistical Package for the Social Science*s* (SPSS), version 11.0.

This study respected the ethical principles, and was approved by the Research Ethics Committee of Universidade Estadual de Montes Claros, report n. 473.371.

## RESULTS

Based on the set of households visited, we collected information referring to 545 children. The loss index was minimum. As to the age of mothers, 48.5% (95%CI 43.9-52.3) were aged from 25 to 34 years, 49.5% (95%CI 45.4-53.7) referred having brown skin, 77.1% (95%CI 73.3-80.4) were married and/or lived in a stable and 26.2% (95%CI 22.2-29.5) studied until elementary school. More than half of the mothers interviewed (54.7%) lived with family income lower than two minimum wages (95%CI 41.7-50.1), as demonstrated in Table 1. Regarding the variables related with maternal and infant health, there were higher frequencies for beginning of prenatal care before the 14^th^ week of pregnancy (78.5%; 95%CI 72.0-79.2), normal delivery (57.7%; 95%CI 53.1-61.3), male child (53.9%; 95%CI 49.6-57.9) and weight at birth ≥ than 2500g (90.5%; 95%CI 7.3-12.3). Most children were followed-up in public health services (73.8%; 95%CI 69.9-77.3), which can be verified in Table 2.

**Table 1:** Socioeconomic and demographic characteristics of children aged less than 24 months. Montes Claros (MG), 2015.

	n	%	95%CI
Maternal age (years)			
19 to 24	204	37.8	33.5-41.6
25 to 34	262	48.5	43.9-52.3
≥35	74	13.7	11.0-16.7
Child gender			
Male	293	53.9	49.6-57.9
Female	251	46.1	41.9-50.2
Mother’s skin color			
Brown/black/yellow	316	58.4	53.8-62.1
White	225	41.6	37.2-45.5
Maternal schooling (years)			
9 (Elementary)	140	26.2	22.2-29.5
≥10 (> Elementary)	393	73.8	68.2-75.7
Mother’s marital status			
Single/Widow	125	22.9	19.6-26.6
Married/stable union	420	77.1	73.3-80.4
Maternal occupation			
Does not work outside the household	366	67.2	63.1-71.0
Works outside the household	179	32.8	29.0-36.9
Number of children			
1 to 2	431	79.1	75.5-82.3
≥3	114	20.9	17.7-24.5
Family income (in minimum wages*)			
<2	250	54.7	41.7-50.1
≥2	207	45.3	34.0-42.1

*Current minimum wage: R$ 724; 95%CI: 95% confidence interval.

**Table 2:** Characteristics related with the health of mothers and children. Montes Claros (MG), 2015.

	n	%	95%CI
Beginning of prenatal care (weeks)			
<14	413	78.5	72.0-79.2
From 14 to 27	101	19.2	15.5-22.0
>27	12	2.3	1.3-3.8
Type of delivery			
Normal	312	57.7	53.1-61.3
C-section	229	42.3	37.9-46.2
Weight-at-birth (g)			
<2,500	52	9.5	7.3-12.3
≥2,500	492	90.5	87.5-92.5
Health service			
Public	402	73.8	69.9-77.3
Private	143	26.2	22.7-30.1

95%CI: 95% confidence interval.

In the analysis of the participation of the child in programs of dietary supplement including ferrous sulfate and vitamin A, indicated by the Ministry of Health, the indexes were 61.0% (95%CI 8.8-14.1) and 67.0% (95%CI 9.8-15.3), respectively.

**Figure 1:**
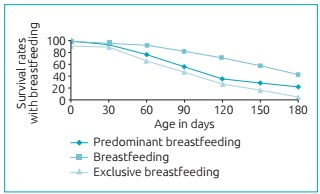
Function of survival rates for breastfeeding, predominang breastfeeding and exclusive breastfeeding in children aged less than 24 months. Montes Claros (MG), 2015.

Figure 1 presents the survival rate curves of maternal breastfeeding for the first six months of life. After 180 days of life, 4.0% of the children received AME; 22.4%, PBF; and 43.4%, complementary maternal breastfeeding (CMB)

**Figure 2:**
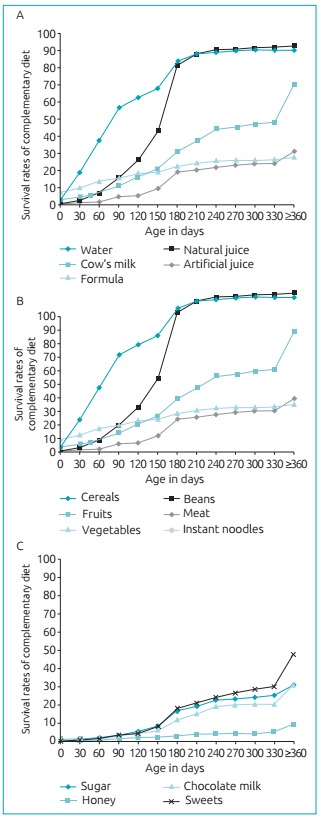
Function of survival rates for the complementary introduction of foods: (A) liquids; (B) solid and semi-solid foods, and (C) junk food in children aged less than 24 months. Montes Claros (MG), 2015.

Figure 2 shows the time of introduction of the three great food groups among the children in the study. In the analysis of liquids, by the third month of life they were given water (56.8%), natural juice/formula (15.5%) and cow’s milk (10.6%). The artificial juice was offered to 31.1% of the participants in the study at the age of 12 months.

Regarding solid and semi-solid foods, cereals, vegetables, beans and meat were introduced at the age of six months in approximately half of the children. Fruits were offered earlier to 45.0% of the participants, at the age of five months. Before completing one year, 25.0% had consumed instant noodles.

As to the analysis of junk food consumption, it was observed that approximately half of the children had already tried sweets (lollypops, candy and caramels) before one year of life. In the same period, sugar and chocolate milk were introduced to approximately 30.0% of the children, whereas 10.0% tasted honey for the first time.

## DISCUSSION

In this population-based study, it was possible to identify the diet of children aged less than 24 months in the city of Montes Claros, Minas Gerais. In the period of AME, children were given water and non-maternal milk, and in the introduction of complementary foods, the introduction of junk food was early. These results reflect the need to implement public policies, considering that actions to promote a healthy diet in childhood have repercussions in the health profile of the population.^3^ ^,^ ^4^ ^,^ ^5^

The National Survey on Demography and Health (PNDS)^10^ assessed the tendencies of breastfeeding in the country and identified the prevalence of 13.2% of PBF in children aged less than six months. Similar results were also obtained in a study in the state of Paraná,^11^ in which the prevalence of PBF in this age group was 11.1%. Among the children in the analyzed city, the practice of PBF overcame these estimations (22.4%), reflecting worse levels of AME. The result demonstrates that even though all mothers breastfed their children, only few did it exclusively until the age of six months.

The frequency of CMB in this study was similar to that observed in a previous national study, which points to this practice in 40.1% of the children aged less than six months. It almost always reflects the early introduction of other types of milk in infant diet to the detriment of AME.^12^

The offer of food before the six months of life causes damage to the infant’s health. However, many mothers believe that liquids, such as juice and other types of milk, can complement maternal milk, providing more energy and nutrients to the infants.^13^

In this population, it was observed that children were exposed to non-maternal milk at the age of three months. The findings are in accordance with the results found by Bortolini et al.,^12^ Schincaglia et al.^13^ and Coelho et al.,^14^ who verified that the intake of other types of milk is high among children aged less than six months; those being breastfed do not need to receive other types of milk or dairy products.^12^

The complementation of the maternal milk with non-nutritional drinks, such as water and teas, is not recommended before the age of six months. In a study conducted in the Southeast region^15^ it was observed that, at the age of 90 days, 23.6% of the children consumed water. Higher estimations were observed in this study, with almost twice as many children drinking water at the same age group. The early intake of foods other than maternal milk by children aged less than six months is strongly influenced by the region. The North of Minas is hot, with climactic conditions similar to those in the North and Northeast regions of the country, where a high consumption of liquids before the adequate period is observed.^16^ This practice is spread because the mother is afraid that, especially in the summer, on hot days, the child may be dehydrated; that is why liquids are believed to be necessary for infants.^17^ The early introduction of water and teas may contribute with the interruption of AME.^14^

The Ministry of Health recommends^18^ the introduction of foods after the sixth month of life. In this stage, maternal milk is not sufficient for the nutritional demands, and complementary foods are essential to provide energy and micronutrients such as iron, zinc, phosphorus, magnesium, calcium and vitamin B6. In developing countries, complementary foods are still a challenge for the good nutrition of children.^19^ ^,^ ^20^ Data from the National Health Survey (PNS), conducted in Brazil in 2013, show high prevalence of unhealthy dietary behaviors in childhood.^21^

The introduction of cereals, vegetables, bean and meat among the studied children is within recommended parameters. However, fruits were offered before the sixth month of life. In a study in the Northwest region of Goiânia,^13^ it was observed that, in the sixth month of life, children already consumed fruits (62.7%), juices (57.2%), and savory foods (55.1%). In the population of Campinas, in the countryside of São Paulo,^14^ 33.1% of the mothers reported offering savory baby food with vegetables, greens and meat from the sixth to the seventh month.

This study showed the early introduction of ultra-processed foods in the infant’s health, which is an inadequate practice in the first years of life and reflects the contemporary diet pattern. These results corroborate data from the literature according to Longo-Silva et al.^22^, who assessed children aged from zero to 36 months in public daycare facilities, identified that industrialized juice was consumed before the first year of life by more than half of the participants, and 10.0% did so before the age of six months, showing the introduction of these items in the diet of infants inadequately and early. Besides, instant noodles were present in expressive frequencies in the diet of the assessed participants in the first 12 months of life. This same result was found by Martins et al.,^23^ who observed the intake of instant noodles in the diet of infants, considered to have risk at birth before the age of six months.

The intake of foods with high concentration of sugars and fats is associated with the occurrence of excess weight and cavities in children.^19^ In this sense, in the first years of life, sugar, coffee, canned food, soft drinks, candy, chips, and other junk food should be avoided.^24^ This study identified that approximately 50.0% of the children before the age of one already ate candy. Corroborating these results, a national study that investigated children in the Brazilian capitals and in the Federal District showed that the introduction of cookies/chips was of 71.7% in the range of nine to 12 months, which is particularly worrying in the South region, where the consumption reached 57.9% among participants aged from six to nine months.^25^ Otherauthors^9^ also observed high intake of fried foods, soft drinks, sweets, junk food and salt in childhood. Strategies in the national scenario have been implemented to improve these rates of quality in infant childhood. The Ministry of Health reformulated the public policies in the field and has recently launched the National Strategy for the Promotion of Maternal Breastfeeding and Healthy Complementary Food in SUS - *Estratégia Amamenta e Alimenta Brasil* (EAAB), which aims at qualifying the work process of basic care professionals, with the objective of reinforcing and encouraging the promotion of breastfeeding and healthy eating habits for children aged less than two years in the Unified Health System (SUS) environment.^26^ In this study, even though most children attended the public health service, the eating practices were inadequate, which suggests the need to improve the actions in these services.^21^ ^,^ ^27^

In this study, the representative sample of children from a municipality placed in a poor region, in which nutritional disorders in childhood constitute a reason of concern, stands out. The data are relevant for the current mother-child health scenario in the context of the studied region (North of Minas Gerais), which is a transition area between the South/Southeast and the Northeast of the country, in order to explore the regional aspects of eating habits in childhood. This information can stimulate local managers and health professionals to propose effective measures of intervention to change the presented scenario.

The results, however, should be interpreted considering some limitations of the research. The retrospective data collection for some variables may be subject to memory bias. Besides, the difficulty of approach regarding diet in epidemiological studies is a limiting factor that should also be considered.

## CONCLUSION

It is concluded that the introduction of complementary foods was early for liquids, honey, sugar and junk food, close to the adaptation for solid and semi-solid foods, which can affect directly the success of breastfeeding. It is possible that the inadequate dietary practices identified can compromise the health of the child in the short and long terms; therefore, it is necessary to prioritize activities to promote and improve mother-child services to change this scenario. In this sense, health professionals play an important role in the counselling of families regarding eating habits in the first year of life, reinforcing the superiority of breast milk and discouraging the introduction of other types of milk, besides mentioning the correct introduction of complementary foods. Further studies are necessary to approach the inter-relations between the variables that interfere in the practice of childhood eating habits.
